# One Health Approaches to Ethical, Secure, and Sustainable Food Systems and Ecosystems: Plant-Based Diets and Livestock in the African Context

**DOI:** 10.3390/foods15010085

**Published:** 2025-12-26

**Authors:** Elahesadat Hosseini, Zenebe Tadesse Tsegay, Slim Smaoui, Walid Elfalleh, Maria Antoniadou, Theodoros Varzakas, Martin Caraher

**Affiliations:** 1 Department of Chemical Engineering, Payame Noor University, Tehran 19395-4697, Iran; hosseinielahe14@gmail.com; 2 Department of Food Science and Post-Harvest Technology, College of Dryland Agriculture and Natural Resources, Mekelle University, Mekelle 231, Ethiopia; ztlovewith73@gmail.com; 3 Laboratory of Microbial and Enzymes Biotechnology and Biomolecules (LMEBB), Centre of Biotechnology of Sfax (CBS), University of Sfax-Tunisia, Road of Sidi Mansour Km 6, P.O. Box 1177, Sfax 3018, Tunisia; slim.smaoui@cbs.rnrt.t; 4 Department of Biology, College of Science, Imam Mohammad Ibn Saud Islamic University (IMSIU), Riyadh 11623, Saudi Arabia; wbelfallah@imamu.edu.sa; 5 Dental School, National and Kapodistrian University of Athens, 11527 Athens, Greece; mantonia@dent.uoa.gr; 6 Department of Food Science and Technology, University of the Peloponnese, Antikalamos, 24100 Kalamata, Greece; 7 Centre for Food Policy, City St George’s, University of London, Northampton Square, London EC1V 0HB, UK; m.caraher@citystgeorges.ac.uk

**Keywords:** sustainability, ethical food systems, One Health approach, plant-based diets, livestock, Africa

## Abstract

The contribution of members of the agri-food system to achieving the Sustainable Development Goals is a key element in the global transition to sustainable development. The use of sustainable management systems supports the development of an integrated approach with a spirit of continuous improvement. Such organization is based on risk-management tools that are applied to multiple stakeholders, e.g., those responsible for product quality, occupational health and safety, and environmental impact, thus enabling better global performance. In this review, the term “ethical food systems” is used in our discussion of the concrete methods that can be used to endorse fairness and concern across the food chain. This comprises safeguarding equitable access to nutritious foods, defending animal welfare, assisting ecologically accountable production, and addressing social and labor justice within supply chains. Ethical factors also include transparency, cultural respect, and intergenerational responsibility. Consequently, the objective of this review is to address how these ethical values can be implemented within a One Health framework, predominantly by assimilating plant-based diets, developing governance tools, and resolving nutritional insecurity. Within the One Health framework, decoding ethical principles into practice necessitates a set of concrete interventions: (i) raising awareness of animal rights; (ii) distributing nutritional and environmental knowledge; (iii) endorsing plant-based food research, commercialization, and consumption; (iv) development of social inclusion and positive recognition of vegan/vegetarian identity. At the same time, it should be noted that this perspective represents only one side of the coin, as many populations continue to consume meat and rely on animal proteins for their nutritional value; thus, the role and benefits of meat and other animal-derived foods must also be recognized and discussed. This operational definition provides a foundation for asking how ethical perspectives can be applied. A case study from Africa shows the implementation of a sustainable and healthy future through the One Health approach.

## 1. Introduction

Different definitions of sustainable food systems include different pillars, such as food security and nutrition, i.e., the achievement of food security and nutrition security through sustainable and secure food systems [1]. Sustainable diets are another significant pillar, targeting low environmental impacts, contributing to food and nutrition security, biodiversity, and ecosystems, and which are culturally acceptable, accessible, economically fair, and affordable [2]. Healthy diets are linked to planetary boundaries. It is key to link sustainable growing practices with sustainable and healthy eating—this is what Lang and Mason refer to as eco-nutrition [1].

Another direction of focus emphasizes sustainable development, prioritizing Sustainable Agriculture and Rural Development (SARD) in the agriculture, forestry, and fisheries sectors. This approach conserves land, water, plant, and animal genetic resources, ensuring environmental non-degradation, technical appropriateness, economic viability, and social acceptability.

The core dimensions of Sustainable development (Economic, Environmental, and Social) are complemented by other dimensions, such as the food, health, nutrition, and cultural dimensions. Here, emphasis is placed on environmental processes and outcomes that can be achieved through sustainable food systems, supporting us in achieving good nutrition and socioeconomic outcomes while keeping environmental impacts suitably low. The aim is not to destabilize the planetary boundaries of biophysical processes—and, hence, environmental systems—but to conserve “natural resources and protect global ecosystems for human and planetary health and wellbeing” [2].

Economic security is another essential pillar of food systems, since they need to achieve economic security for all stakeholders and combat existing malnutrition-/obesity-related health problems [3]; these problems represent the triple burden of malnutrition which includes undernutrition, overnutrition, and micronutrient deficiencies. Social and cultural elements related to food consumption are also addressed by taking into account resilient and equitable food systems, sociocultural wellbeing, social system integrity, the right to food, the right to freedom from poverty and inequality, the right to inclusion, the support of the common good, and the prioritization of just, ethical, and equitable food systems [4].

Livestock perform an important role in global food security, particularly in areas where crop-based diets alone cannot meet nutritional needs, or where climatic factors and soil fertility limit crop production [5,6]. Ruminants have the exclusive ability to change plant materials which are indigestible to humans—like crop residues [7,8], low-quality forage [9], and agricultural byproducts—into high-quality whole proteins that are abundant in micronutrients and essential amino acids [10]. This adaptation not only improves human nutrition but also exploits the use of agricultural resources that would otherwise be wasted [11,12,13]. Beyond meat and milk, livestock produce a wide array of valuable byproducts, counting hides, wool, gelatin, and materials employed in industrial and food-processing applications, contributing further to economic value and resource competence [14,15]. In addition, livestock systems can support the productive employment of marginal lands that are unsuitable for conventional crop farming [16]. Grazing in these parts does not have a large impact on arable land and, in many regions, such as sub-Saharan Africa, it supports local livelihoods, conserves cultural traditions, and preserves ecosystem biodiversity [17,18,19]. Also, livestock serve a pivotal role in organic waste management, changing crop surpluses, food processing residues, and other organic byproducts into nutrient-rich manure, which develops soil fertility and decreases environmental pollution. Moreover, integrated livestock–crop systems can contribute to carbon sequestration and sustainable land management when properly managed [20]. Overall, sustainable livestock management represents a multifaceted approach that combines nutritional security, efficient resource utilization, environmental stewardship, and socioeconomic resilience. By connecting animal production with ecological and human health objectives, livestock remain a central component of resilient and sustainable food systems worldwide. Actually, cultured (laboratory-grown) muscle cell (CMC) food has emerged due to pressure on traditional food systems and the negative aspects of livestock production [21]. The CMC industry claims to reduce the demand for natural resources, have a minimal impact on the environment, and ensure better outcomes for animal welfare [22,23,24]. CMCs are globally controlled by a small number of companies, making the transfer to small- and medium-sized farmers/growers difficult; they are currently not market-sellable due to high start-up costs. Moreover, livestock production systems should be discussed and considered within the framework of One Health (https://commission.europa.eu/publications/sustainable-europe-2030_en, accessed on 15 December 2025). One Health prioritizes healthy animals, healthy ecosystems, and healthy people [25]. The adoption of agroecology is another factor in the transformational change in livestock industries [26]; it necessitates the combination of livestock production with ecosystem components, including water filtration, nutrient cycling, soil carbon, and increased biodiversity. It is possible to scale agroecology and equally apply it at the levels of both smallholder farmers and large livestock enterprises [27,28] (https://www.ilri.org/news/one-health-key-sustainable-livestock-and-human-and-environment-health, accessed on 15 December 2025). Healthy ecosystems with ethical and sustainable production of natural animal proteins depend on the complementarity of One Health and agroecology [29,30,31].

Moreover, animal welfare is recognized as a critical concern by the UN’s One Health Expert Panel and other global stakeholders [32]. However, it has not been considered in key climate discussions, such as those presented in the Intergovernmental Panel on Climate Change (IPCC) Sixth Assessment Report. The latter fails to address the One Health framework or the welfare implications of mitigation strategies, with priority being placed instead on reducing emissions from animal agriculture [33].

**Figure 1:**
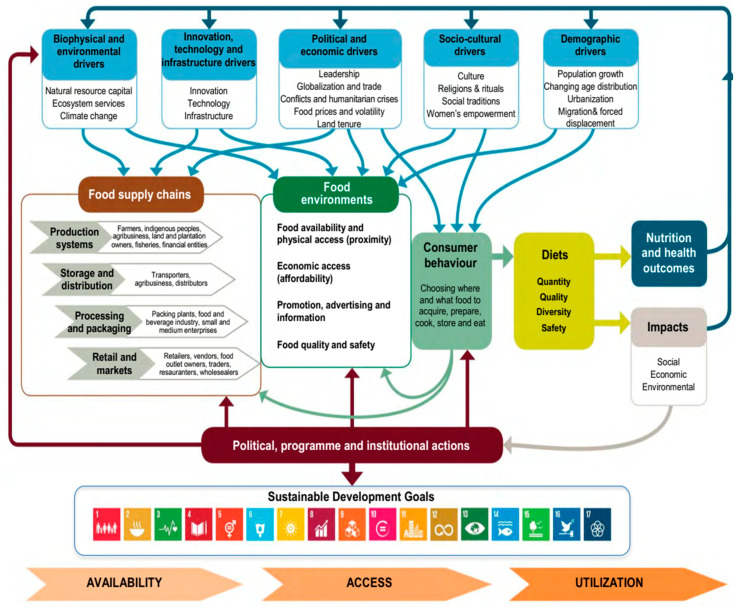
Sustainable food systems conceptual framework [34].

Despite the increasing recognition of these sustainability pillars, ethical consumption, and the One Health perspective, the integration of these dimensions into a unified framework guiding food system transformation remains limited. In particular, the intersection of plant-based dietary shifts, governance tools, and regional case studies (such as Africa) has not been sufficiently explored. The aim of this article is therefore to examine how food systems can be made both sustainable and ethical by incorporating the One Health approach, addressing psychological, social, and structural barriers to dietary change, and presenting strategies and examples that can inform global practice. As shown in Figure 1, there are many components and drives in the sustainable food system framework. It also shows that the food supply chain and food environments are integrated to assure sustainable development goals.

The above conceptual framework is not only descriptive but also strategic as it underlines the systemic nature of food challenges while mapping the linkages across economic, social, environmental, and ethical domains. This integrative perspective provides the basis for identifying governance tools, consumer-oriented interventions, and dietary shifts, elements that will be further elaborated in the following sections.

In this review, the term “ethical food systems” refers to practical approaches that promote fairness and responsibility across the food chain. This includes ensuring equitable access to nutritious foods, protecting animal welfare, supporting environmentally responsible production, and addressing social and labor justice within supply chains. Ethical considerations also encompass transparency, cultural respect, and intergenerational responsibility. This operational definition provides a foundation for how ethical perspectives are applied throughout the review.

## 2. Methodology

This review adopted a narrative synthesis approach due to the multidisciplinary and conceptually heterogeneous nature of ethical food systems, sustainability frameworks, One Health principles, and dietary transitions. The literature spans empirical studies, policy analyses, nutrition science, environmental assessments, and socio-behavioral research, rendering a meta-analytic or strictly systematic design unsuitable. Narrative synthesis supported the integration of diverse evidence sources while preserving contextual depth and conceptual coherence across plant-based dietary frameworks, livestock sustainability considerations [4,5,6,7,8,9,10,11,12,13,14,15,16,17,18], and One Health approaches relevant to African regions [28,29,30]. This methodological choice is aligned with international recommendations for complex, system-oriented research, where interactions between environmental, nutritional, ethical, and sociopolitical dimensions must be jointly interpreted.

Relevant peer-reviewed articles, policy documents, and international reports published between 2015 and 2025 were identified through structured searches in Scopus, Web of Science, and PubMed, complemented by targeted explorations of official sources such as FAO, the WHO, the IPCC, and the European Commission. To increase transparency, the search strategy followed an iterative multistage process. First, an initial set of keywords was used (“sustainable food systems,” “One Health,” “ethical consumption,” “plant-based diets,” “livestock sustainability,” “agroecology,” and “Africa case study”). Keyword combinations were subsequently expanded through backward and forward citation tracking to capture foundational and emerging research across environmental, nutritional, ethical, and policy domains. No study design or country restrictions were applied, given the narrative nature of the review and the need to accommodate diverse methodological approaches.

To minimize thematic and interpretive bias, particularly regarding potential imbalances between plant-based perspectives and livestock-related evidence, the review employed an intentional dual-domain inclusion strategy. Studies addressing sustainable livestock production, resource-efficient feeding, and the conversion of agricultural byproducts into high-quality animal protein [6,7,8,9,10,11,12,13,14] were included alongside those supporting plant-based transitions and environmental impact mitigation. Screening and thematic categorization were performed independently by all co-authors, followed by cross-validation to resolve discrepancies. This balanced inclusion strategy responded directly to concerns that ethical and environmental narratives may overemphasize plant-based outcomes while underrepresenting the multidimensional role of livestock within food security, rural economies, and One Health frameworks. Overall, studies were eligible for inclusion when they met one or all of the following criteria: (i) presented conceptual frameworks related to sustainability, ethics, or One Health; (ii) provided empirical evidence on plant-based diets, livestock systems, environmental impacts, or dietary transitions; (iii) offered policy analyses relevant to food system governance. Peer-reviewed and authoritative institutional documents were prioritized, while the grey literature was included only when it provided essential contextual insights. Furthermore, publications were excluded if they lacked clear linkage to these intersections, were purely technical without conceptual relevance, or did not contribute to the integrative synthesis required for a One-Health-oriented food system perspective. Finally, records removed for other reasons were those that were non-English publications without translations, were inaccessible in their full text, and highly technical articles without conceptual relevance to ethical or One Health frameworks. Particular attention was paid to identifying potential biases, such as industry influence on dietary recommendations or limited geographic coverage in empirical studies. The screening process involved independent evaluation by all co-authors, with discrepancies resolved through discussion and consensus.

**Figure 2:**
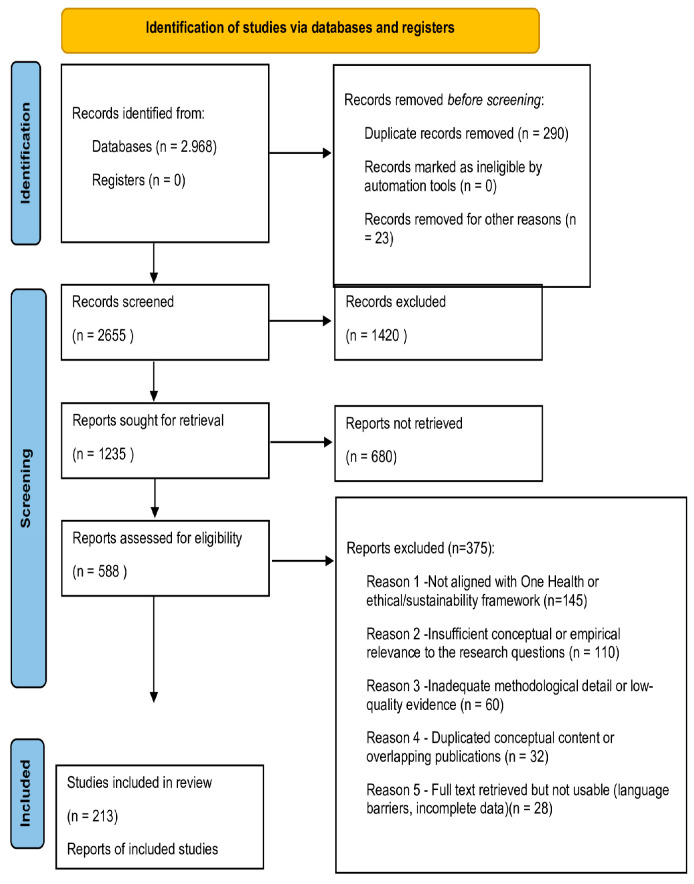
PRISMA 2020 flow diagram illustrating the identification, screening, eligibility assessment, and inclusion of sources in this narrative review.

Although this review follows a narrative design, an adapted PRISMA-style flow diagram was incorporated to enhance transparency in the identification, screening, and inclusion processes (https://www.prisma-statement.org/prisma-2020-flow-diagram, accessed on 15 December 2025). The final synthesis included 180 publications spanning peer-reviewed studies, policy frameworks, and institutional reports. Figure 2 depicts a PRISMA 2020 flow diagram illustrating the identification, screening, eligibility assessment, and inclusion of sources.

## 3. Addressing Food Systems Complexities and Ethical Food Consumption

Different tools can be used to address food system complexities. Traditionally, the impact of refrigerated chains on food conservation and environmental costs [35], the implications of emerging food technologies, such as plant-based and cultured meat [36], and the effects of alternative diets on systemic outcomes [37,38] could affect these complexities [39]. However, there is concern that national-scale tools might reduce their effectiveness in capturing on-the-ground barriers to food access and consumption, and fail to correlate these local dynamics to environmental, social, economic, and health dimensions. In addition, even though supply chains are affected by consumer demand for fair, healthy, and sustainable food production, local tools frequently overlook diets and consumer behavior [40].

The intersection of food security and nutrition with ecological, health, economic, political, and sociocultural systems and the demand for healthy interrelationships for long-term food system viability are reported clearly by Clapp et al. [41], linked to the right to food. Sustainable diets can also be associated with the right to adequate food, as indicated in many studies [42,43]. It is important to note that the way in which that right to food is defined determines whether a consumer or a citizen is seen to have their rights met [44]. The right to food security is a fundamental human right ensuring physical and economic access to sufficient, safe, nutritious food for a healthy, dignified life. This right encompasses availability, accessibility, adequacy, and sustainability and is legally recognized by the UN (ICESCR Article 11). Hence, governments should respect, protect, and fulfill this right. The transformation of the entire food system will affect diet improvement, affecting the global burden of disease; this is also known as the diet-focused perspective [45].

Sustainable development in terms of its core dimensions is often associated with cultural aspects, food quality, health and nutrition, and governance [46]. The privatization of carbon markets, corporate-driven regenerative agriculture, and technological fixes are all responses to the challenges posed by unsustainable food systems. In this context, structural inequalities are avoided, resulting in ‘greenwashing’ and ‘social washing’ practices [47]. However, the question of whether these inequalities are avoided or reinforced has been addressed. Carbon footprints or yield efficiency are examples of an oversimplification of sustainability; with issues such as poverty reduction, inequality, hunger, and violations of community livelihoods being neglect-ed. 

Different systems, such as Global Positioning System(s) (GPS(s)), have been employed to define exposure to the food environment. In this context, ‘exposure’ generally refers to an individual’s spatial and temporal proximity to food outlets, such as the number, density, or types of outlets encountered within a person’s GPS-tracked activity space or along daily routes. Using such GPS-based measures, Siddiqui et al. [48] examined associations between food-outlet exposure and diet-related or cardiometabolic outcomes, but no consistent relationships were identified. Very simply mobile phone technologies can enable growers to check weather forecasts, download apps to help them in planning the growth of their crops, and even to check market prices before selling.

Ethical decision making in food consumption is essential, as reported by Varzakas and Antoniadou [49]; this can be enhanced by new tools, such as digitalization, which will enhance consumers’ decision making and affect food systems, as reported by Jose et al. [50]. The digitalization of livestock farming will aid in the monitoring of the health, welfare, reproduction, and environmental impact of individual animals in real-time [51]. New and traditional technologies such as Radio Frequency Identification (RFID), infrared thermal imaging [52], audio surveillance systems [53], deep learning and image analysis [54], two- and three-dimensional cameras [55], and accelerometers can be employed to monitor and manage animal welfare. Food systems face food and nutritional insecurity, along with climate-change-related challenges and unsustainable farming practices [56]. Emerging digital technologies, such as artificial intelligence (AI) and a subset of it, i.e., machine learning (ML), have been found to mitigate these challenges [57].

Prioritization of attributes such as animal diets, stress-free environments, humane processing practices, and health conditions, linked to both ethical and hedonic values, has been indicated by consumers. Some studies also describe a potential solution to global food insecurity in GMOs; these provide a modern, efficient solution, overshadowing their socioeconomic and environmental risks [58]. With plant-based development, it is not GMOs but genomic editing or CRISPR that are key. CRISPR seems to be more acceptable to the public than GMOs, as it does not involve cross-species transfer.

However, other studies highlight that, when effectively regulated and embedded within supportive policy frameworks, GMOs can also enhance the resilience and economic viability of small-scale farmers by improving their resilience against drought, pests, and disease [59,60]. Are GMOs a symbol of progress, or do they marginalize alternative models like agroecology by labelling them as inefficient or unscientific [61]?

GMOs might displace small farmers and lead to the disruption of local food systems and the consolidation of land ownership among corporations [62]. Emphasis on the preservation of traditional seeds, biodiversity, and agroecological practices as being able to restore autonomy over food systems has been discussed by community-led resistance movements, such as those in Ghana and Argentina [63]. Raimi and Masri [64] applied critical discourse analysis (CDA) to analyze how GMO agripreneurship impacts the global food supply chain. They proposed agroecological sovereignty as a hybrid approach that balances decolonization.

In sum, ethical food consumption is increasingly being shaped by digital technologies, consumer values, and contested innovations such as GMOs. These dynamics reveal both opportunities for more transparent and welfare-oriented food systems and risks of reinforcing inequalities or displacing traditional practices. Recognizing these tensions provides a foundation for the following sections, where the focus shifts from individual consumption choices to broader governance mechanisms and the systemic transformations needed to ensure sustainability and equity across food systems.

Moreover, we should point out that the negative perception of livestock production along with the concern of livestock health has led to discussions about reducing meat consumption or even eliminating it from diets [65,66,67,68]. Of course, it is important that we do not forget the growing global conversation in the framework of One Health for the development of livestock production systems (www.commission.europa.eu/publications/sustainable-europe-2030_en, accessed on 15 December 2025), considering healthiness of animals, ecosystems, and people [25]. The example provided by agroecology should also be mentioned, as it brings together livestock and ecosystem services [26,69,70]. Pasture-based grazing systems will be included within this agroecology. However, whether nutritious and natural meat will be produced, and whether diseases will occur, remain to be seen.

Finally, despite the benefits of natural agrifood systems, one cannot ignore the anthropogenic greenhouse gas emissions released by the livestock industry [71]; it is also important to note the efforts that are being made to reduce the impact of livestock on the environment through grazing land management [72] and ruminants’ enteric methane production [73]. Additionally, the use of foodstuffs such as soy for animal feed as opposed to their being used for human consumption represents an inefficiency in energy transfer.

## 4. Advancing Nutritional and Environmental Awareness

Nutrition affects both health and the environment, influencing climate change, biodiversity loss, and food security [74]. Despite the information available, mixed messages lead many to consume high amounts of animal products, primarily from cows and sheep, and processed foods, which harm ecosystems [75,76]. However, such mixed messages are less evident among countries in the European Union, where the Farm to Fork Strategy outlines a policy direction toward healthier and more sustainable diets, and where official dietary guidelines explicitly recommend reducing meat consumption and increasing plant-based foods for both health and environmental reasons [77,78]. In 2015, the US Dietary Guidelines Advisory Committee (DGAC) recommended more plant-based and fewer animal-based foods for health and environmental benefits, but this was excluded from the final USDA guidelines [79,80]. Sharing knowledge through trusted experts can link health benefits to environmental care, motivating sustainable choices [76]. Public awareness, supported by education, media, and policy, is essential for advancing sustainable food systems [81].

### 4.1. Key Methods of Knowledge Dissemination in Nutrition and Sustainability

Spreading knowledge about healthy eating and its environmental impact requires varied approaches, reaching people of all ages via education, media, policies, and clear science communication to encourage better choices and support of sustainable food systems. Integrating sustainability and nutrition into school curricula effectively raises awareness. However, digital platforms may also circulate misleading or sensationalized nutrition messages, which can create confusion among audiences and complicate effective communication [82]. Nutrition educators should promote healthy diets and ecological awareness [76]. Schools can foster this by combining biology, environmental science, and health education with hands-on activities like gardens, plant-based cooking, and sustainable lunch programs. Given the link between animal-based diets and environmental harm, addressing ecological impacts in curricula is vital [80].

### 4.2. Media and Digital Platforms: Social Media, Documentaries, and Awareness Campaigns

**Figure 3:**
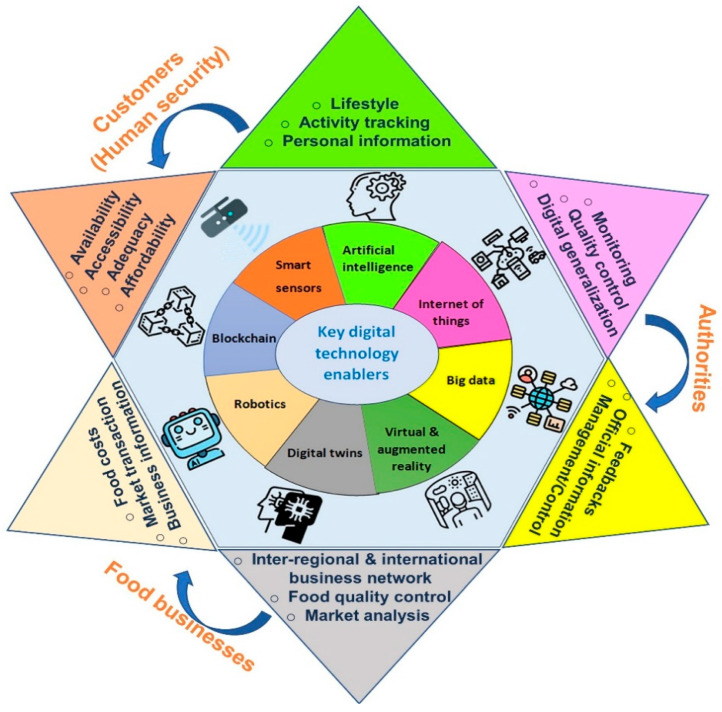
Food system and digital transformation [86].

Social media platforms like Instagram, YouTube, TikTok, and X have become influential in linking eating habits with personal and environmental health, with plant-based chefs, health professionals, and science communicators promoting critical thinking about food choices [83]. Documentaries such as Cowspiracy, What the Health, and The Game Changers show that storytelling can make scientific information more accessible [84]. Digital tools like carbon footprint calculators, sustainability labels, and green recipe apps further help individuals assess the environmental impact of their diets [85]. With over half of Americans concerned about climate change [76], interest in diet advice that supports the environment is growing. Digital platforms thus play a key role in promoting healthier, more sustainable eating, a point highlighted in the 2015 DGAD report [79]. Industry 4.0 technologies like AI, big data, IoT, and blockchain are transforming agriculture and food systems toward sustainability [75]. Figure 3 shows key components of this digital transformation.

### 4.3. Public Policies and Food Labels: Helping People Make Greener Choices

Public policies and clear food labeling are essential for promoting sustainable habits. Labels that show resource use or product origin help consumers make eco-friendly choices, and recognized labels like EU Organic, Rainforest Alliance, and Carbon Trust can be used to guide buyers. The EU plans a unified sustainability label covering the full product lifecycle, complementing existing ones [87]. Studies on olive oil show that combining EU Organic and Sustainable Irrigation labels improves consumer perception and purchase intent [88]. The Rainforest Alliance label supports sustainable farming and social aspects, but faces communication challenges [89]. The Carbon Trust label reveals products’ greenhouse gas emissions, aiding carbon footprint comparisons and raising awareness [90,91]. The SNEB supports sustainability in national dietary guidelines (3). Countries like Brazil already have such practices in place, influencing public and institutional purchasing [79,92]. Clear, standardized, science-based labeling with strong regulation is needed for practical impact.

### 4.4. Scientific Research and Public Engagement: Bridging the Gap Between Academia and the Public

Scientific research is the basis for reliable advice on nutrition and environmental sustainability. Studies consistently show that animal-based foods (especially from ruminant animals) produce more greenhouse gases and use more land and water than plant-based alternatives. In this regard, tools like Life-Cycle Assessment (LCA) confirm these impacts [76,93]. Experts thus recommend increasing consumption of whole grains, legumes, fruits, and vegetables [94]. However, research must be communicated clearly and accessibly. Researchers are encouraged to engage beyond academia, collaborating with media, educators, policymakers, and communities through methods like citizen science, public talks, open data, and partnerships with digital creators [79]. These approaches align with community-based social marketing, emphasizing clear messaging, local engagement, and behavioral insights to foster sustainable food choices [95]. Table 1 summarizes the key knowledge dissemination strategies discussed in this section, outlining their strengths, intended audiences, and examples from real-world applications.

## 5. Challenges in Spreading Nutritional and Environmental Knowledge

Even though the significance of sustainability and nutrition is becoming widely recognized, several challenges are hindering the effective dissemination of knowledge in this field. These challenges span from conflicting information to deeply rooted psychological and structural barriers. Understanding and addressing these challenges is essential for promoting general behavioral change and policy reform. A big challenge is posed by cultural factors and the perceived role of meat in health, especially among males. Also, meat is associated with affluence among migrant communities who had previously consumed little meat.

### 5.1. The Conflict Between Industry Narratives and Scientific Facts

A key challenge in sharing reliable nutrition and sustainability knowledge is conflicting media messages, with industries often altering or obscuring scientific findings, creating public confusion and distrust. Many commercial websites mix accurate and false information, while government and academic sources are generally more reliable [96]. This confusion undermines confidence and makes it harder to identify truly healthy and sustainable foods. Building a society that values health and trustworthy information, supported by clear public understanding, is essential for guiding better dietary choices.

Another structural driver of mixed messages is the profit-maximization logic within the food industry, which strongly promotes ultra-processed foods (UPFs) despite their poor nutritional quality. UPFs are inexpensive to produce and heavily marketed, making them far more accessible than healthier alternatives, especially for lower-income consumers who may be unable to switch even when they intend to. This creates economic and informational pressures that reinforce unhealthy dietary patterns and highlight the need for stronger systemic responses to protect both consumers and planetary health [97].

### 5.2. Psychological Obstacles to Dietary Change

Despite expert support for healthier, more sustainable diets, many struggle to change due to cultural, emotional, and habitual factors. Even when aware of plant-based benefits, people often resist change. Habit is key; many enjoy meat, are used to it, and lack knowledge about plant-based alternatives, making shifts difficult [98]. Emotions and culture, including comfort and tradition, also play roles. Attachment to familiar foods, distrust of new advice, and limited professional support cause people to ignore facts and maintain their usual diets [99]. Perceived costs, complexity, and restricted access to sustainable foods add barriers. Mixed online messages reduce trust in science-based guidance [96]. Overcoming these challenges requires simple, supportive, and relatable messages that show plant-based eating as affordable and easy.

## 6. Economic and Political Influences on Food Systems

Economic and political factors strongly influence food systems. In many countries, meat and processed food production receive more government support than fruits and vegetables, which are classified as “specialty crops” and receive minimal funding [100]. Corporate lobbying can also shape nutrition advice, as seen in the U.S. in 2015, when the DGAC’s recommendation to reduce red and processed meat for environmental reasons was omitted from final guidelines under industry pressure [79]. Support for sustainable farming remains inconsistent, with large industrial farms often receiving substantial subsidies despite environmental harm, while eco-friendly farmers receive less assistance [76]. For instance, in 2020, the U.S. canceled a USD 3 billion climate-smart agriculture program, showing how political decisions can hinder sustainability progress [101]. Disseminating nutritional and environmental knowledge can transform food systems, making them more sustainable and ethically grounded, by fostering critical reflection among consumers, health professionals, and policymakers on the ethical dimensions of food production, consumption, and policy.

## 7. Raising Awareness About Animal Rights and Welfare

Animals in large-scale farming often face poor living conditions, raising serious ethical concerns [76]. Research shows that when people learn about animal suffering and their capacity to feel pain and emotions, they tend to care more about animal welfare [102]. This awareness can lead to negative attitudes toward eating meat and increased interest in plant-based diets. Visual media, such as documentaries and online campaigns, have played a key role in exposing issues like factory farming and slaughterhouse practices, increasing public demand for more humane food systems [103]. However, emotional reactions alone may not create lasting change. Studies suggest that effective media should combine emotional impact with clear guidance for action while considering social norms and possible barriers to change [104].

## 8. Encouraging Plant-Based Diets or Traditional Meat Proteins Through Education and Commercial Promotion

Owing to their high biological value and whole amino acid profiles, meat proteins hold a dominant place in human nutrition [105,106]. Differently from most plant-based proteins, meat covers all essential amino acids in adequate amounts, which makes it extremely significant for growth, the preservation of muscles, immune function, and metabolic health [107,108,109]. Beyond that, meat is a source of numerous micronutrients that are typically restricted in plant-based diets, including vitamin B12, heme iron, zinc, selenium, and long-chain omega-3 fatty acids, which are important for neurological function, cardiovascular health, and immune competence [110]. Nevertheless, notwithstanding these nutritional welfare factors, meat consumption does have risks; high consumption of red and processed meats has been related to an increase in cardiovascular risk, colorectal cancer, and other chronic diseases [111,112]. Therefore, dietary recommendations usually propose restraint of intake. Environmental matters are also important, particularly concerning greenhouse gas emissions, land use, water use, and impacts on biodiversity because of livestock production. However, implementing new traditions of managing livestock production in a more sustainable manner—such as rotating grazing, improving the efficiency of feedstuff use, reprocessing manure, mixing crop and livestock production, and using low-emission technologies—has shown that many of these influences can be achieved without sacrificing nutritional value.

From a food security perspective, livestock transform human-indigestible feedstocks—for instance, crop residues, low-quality forages (either as a result of bad growing conditions or because of the higher yields of crops grown for livestock where emphasis on quality is less important), surplus crops (in good years), and organic waste (including the byproducts of food processing, e.g., citrus pulp, distiller’s grain)—into high-quality protein, thus improving resource efficiency and decreasing food loss. Indeed, without livestock, we would have a serious organic waste disposal problem.

For example, meat production among occupations in rural and marginal areas where crop farming alone is not possible contributes to socioeconomic resilience. In addition, livestock grazing on marginal lands, which are inappropriate for crop farming, can aid the maintenance of ecosystem services such as soil fertility, carbon sequestration, and biodiversity. While plant-based proteins are significant for sustainability and health, meat proteins are a vital part of worldwide nutrition. Meat’s dietary benefits and its environmental and health considerations must be held in balance, allowing us to focus on the integrated approaches that promote sustainable and ethical food systems.

It should also be stressed that livestock (mostly ruminants) makes it possible to feed people in locations where a vegetarian diet would not suffice or is not possible; thus, meat contributes significantly to food security in many locations and contexts. Livestock also produce many valuable byproducts (manufacturing, food processing, animal feed). Moreover, there has been significant progress over time in reducing the environmental impact of livestock.

Meat also provides higher quality protein than plants (1.4:1) and creates 1 high-quality protein unit per 1.4 units of plant protein they consume (meaning the impact is not significant).

One could also add that some land in Africa and many other places is not suitable for crop production but can be used as pastureland; rangeland is inherently more biodiverse than cropland.

Plant-based diets are gaining attention not only for their health and environmental benefits but also for ethical reasons. Learning that plant-based foods generally require less land and water and produce fewer carbon emissions helps people understand the ethics behind their food choices. Rose et al. emphasize that eating fewer animal products and more vegetables, legumes, and whole grains benefits both health and the planet [76]. Animal foods, especially beef, involve the emission of far more greenhouse gases than plant-based foods, with meat producing over 30 kg CO_2_-eq/kg compared to most plant foods, producing under 1 kg [94]. Promotional tools such as “climate-friendly” labels and positive environmental marketing can reduce meat consumption and promote plant-based diets [113]. Tailored strategies with cooking guides, shopping tips, and meal plans, particularly for younger consumers, are more effective when linked to ethical or ecological values [114]. Companies increasingly invest in plant-based products, using in-store campaigns, restaurant partnerships, school collaborations, and product sampling to encourage sustainable eating [115].

## 9. Supporting Agri-Food Reform by Informing Policymakers and Consumers

Scientific research increasingly shapes food policies as environmental concerns grow. Awareness of the high emissions involved in livestock encourages support for eco-friendly farming and climate-focused dietary guidelines [94]. The European Commission recommends making sustainable food options more accessible and appealing [116]. Consumers, equipped with clear information and tools, can influence the market toward ethical choices. However, Reisch notes that effective communication must incorporate behavioral science to truly drive change, empowering individuals and institutions to foster healthier, more resilient food systems [117].

In conclusion, the effective transfer and communication of nutritional and environmental knowledge play a pivotal role in aligning individual behaviors, institutional practices, and policy frameworks with the principles of sustainability and ethics. While education, digital platforms, public policies, and scientific engagement provide powerful vehicles for change, their impact depends on overcoming misinformation, psychological resistance, and structural inequalities. Promoting informed choices and embedding ethical awareness into food systems establishes the foundation for more profound transformations, enabling a shift from awareness-raising toward systemic reforms that will be examined in the following sections.

## 10. Promotion of Plant-Based Food Research, Commercialization, and Consumption

The commercialization of plant-based foods generally follows an adaptive innovation pathway in which scientific knowledge is translated into market-ready products. The early stages focus on idea generation, shaped by consumer expectations, sustainability priorities, and evidence emerging from nutrition and public health research. Rather than emphasizing technical formulation, this phase is driven by understanding societal needs and identifying opportunities where plant-based alternatives can offer environmental or health advantages [118]. In developing plant-based alternatives, the focus has increasingly shifted from purely technical formulation to understanding how consumers perceive taste, texture, and nutritional value. Recent research explores a wide range of plant-derived ingredients to better align these products with the sensory qualities and health attributes associated with traditional animal-based foods. This work reflects broader efforts to balance environmental benefits with consumer expectations, rather than emphasizing the technical specifics of any single ingredient.

For example, current research highlights the growing interest in plant-derived proteins, such as pea protein, which can support the development of appealing plant-based products. Rather than focusing solely on their functional or processing properties, recent studies emphasize their potential to meet consumer expectations for taste, texture, and nutritional adequacy. This shift reflects a broader trend in which ingredient choices are guided not only by technological considerations but also by market demand for accessible and nutritionally meaningful plant-based alternatives [119]. Although plant-derived proteins such as pea protein offer several advantages, their use in commercial plant-based foods is more influenced by consumer-oriented attributes than by processing characteristics. Factors such as perceived health benefits, hypoallergenic properties, and interest in non-genetically modified ingredients have increased their relevance in product development. Recent studies highlight that these proteins are being explored not only for nutritional purposes but also for their potential role in creating more sustainable and environmentally friendly food materials [120]. As a result, pea-based ingredients are now widely incorporated into cereals, baked goods, dairy alternatives, and meat substitutes, reflecting their growing importance in supporting the expansion of plant-based food markets [121].

Algae, fungi and precision fermentation have recently attracted growing attention in the global food and beverage sector, where they are increasingly marketed as nutrient-dense “superfoods” and incorporated into premium product launches as ingredients, natural colorants, or flavor-enhancing components [122]. The commercial momentum behind algal products is reflected in market trends: the global algal products sector was valued at USD 5.3 billion in 2023 and is projected to reach USD 7.3 billion by 2028, with a CAGR of 6.4% [123]. Similarly, the worldwide algal protein market reached USD 3.2 billion in 2021 and is expected to grow at a CAGR of 8.4% through 2030 [124]. Although microalgal protein extracts typically display low solubility close to their isoelectric points, they show high solubility in neutral–basic environments [125,126,127]. These investigations highlight the versatility of algal biomass in food innovation, encouraging its application across snacks, sauces, baked goods, meat alternatives, and desserts [128]. Collectively, these economic and scientific trends indicate that algae may play an increasingly meaningful role in the broader transition toward more sustainable and plant-forward food systems. Michel et al. [129] specified that consumers had favorable thoughts on algal meat analogs because of the environmental and nutritional advantages. Van der Stricht et al. [130] reported that E.U. consumers were unfamiliar with food products containing microalgal proteins. Likewise, Mellor et al. [131] reported that British consumers had limited knowledge of algae as a food source but showed interest in trying them, and the acceptance of algae was determined by the supposed benefits in innovation, taste, wellbeing, sustainability, and affordability. Lafarga et al. [132] pointed out that Spanish consumers viewed microalgae as sustainable, nutritious, and safe; however, low consumption was mainly attributed to a lack of awareness and unfamiliarity. Overall, improving consumer familiarity with algae and algal proteins appears essential for strengthening adoption and market growth. Despite this potential, flavor remains a key barrier to mainstream acceptance, as both aroma and basic taste profiles may differ from those of conventional products.. Addressing these sensory concerns through continued innovation in product design and consumer-centered formulation will be crucial to enhancing product appeal and supporting broader market development.

Once a promising concept is identified, the development process moves toward creating early prototypes. At this stage, the focus shifts from technical formulation to evaluating how well the products align with consumer expectations in terms of taste, texture, and overall appeal. Collaboration across different disciplines—including nutrition, sensory science, and product development—helps refine these early versions and ensure they are suitable for broader testing.

Instead of emphasizing detailed processing steps, the prototyping phase in plant-based innovation is increasingly shaped by consumer-centered evaluations and market insights. Feedback on flavor, quality, and usability guides adjustments before products are introduced to pilot trials or early market testing. These iterative steps help companies to assess whether new products can be scaled effectively and whether they align with evolving consumer preferences. When these indicators are favorable, the product moves toward larger-scale production, distribution planning, and targeted communication strategies. In this way, plant-based commercialization relies on a flexible and adaptive pathway that integrates consumer insight, innovation, and market readiness.

To promote regular plant-based consumption, integrating plant-based options into public institutions and commercial settings is a strategic approach. For instance, New York City has adopted plant-based meals as the primary offering in all public hospitals, resulting in a 36% decrease in food-related greenhouse gas emissions and cost investments of USD 318,000 in 2023 [133]. The platform attained over 90% patient fulfillment, representing the viability and welfare of plant-based blackboards in healthcare settings [134]. Columbia University has committed to the reduction of its food-related carbon emissions by 25% by 2030 as part of New York City’s Plant-Powered Carbon Challenge [134]. This initiative stimulates organizations to drop their carbon footprints by growing plant-based meal aids and reducing emissions from food procurement [135]. In the first year of contribution, Columbia Dining realized a reduction of 1.6% in emissions strength, notwithstanding a 10% increase in meal quantity. Other NYC institutions have also combined the challenge. NYC Health + Hospitals is distinguished for serving over 1.2 million plant-based meals, resulting in a 36% decrease in carbon emissions and a 60% cost savings per meal [134]. In the UK, the “Plants First Healthcare” campaign supports making plant-based meals the default option in National Health Service (NHS) hospitals. Supported by signatories, the campaign underlines that such a change could save GBP 74 million annually and decrease food-related emissions by up to 50% [136].

Table 2 provides a comparative overview of the most established and emerging plant-based food sources, highlighting their primary processing methods, functional applications, commercialization, and consumer acceptance trends. It includes widely used ingredients such as soy, oats, and pea protein, as well as novel and underutilized sources like algae, jackfruit, mycelium, and duckweed. Each source is assessed for its technological readiness and market integration, offering insights into its role in current and future plant-based food innovation. In summary, the commercialization of plant-based foods reflects a dynamic innovation pathway that moves from scientific discovery to large-scale adoption. While ingredients such as pea protein and algae illustrate the technological promise and the sensory challenges of alternative proteins, their integration into institutional and commercial contexts demonstrates tangible environmental and economic benefits. As public and private sectors increasingly invest in scaling plant-based solutions, the combination of scientific advances, consumer acceptance, and supportive policy frameworks will determine the long-term role of these products in sustainable and ethical food systems.

## 11. Fostering Social Inclusion and Positive Recognition of Vegan Identity

Sustainable Development Goal 2, aiming to ensure universal access to safe and nutritious food, resonates with veganism. Sustainable Development Goal 3 focuses on good health and wellbeing, while Sustainable Development Goal 15 is centered around life on land, in connection with vegan diets [150]. Vegan behavior adoption is interlinked with human health, animal welfare, and environmental sustainability, as reported by many authors [151,152,153]. SDGs 14 and 15 refer to life below water and on land, respectively; so, we ask, how does this relate to veganism?

SDG 14 is focused on conserving and sustainably using oceans, seas, and marine resources for development, aiming to combat pollution (especially plastics), acidification, and overfishing, while protecting marine ecosystems and increasing scientific knowledge in support of healthier, productive oceans vital for food, climate, and livelihoods. It covers reducing pollution, protecting marine areas, managing fisheries, reducing acidification, and increasing ocean research, acknowledging the ocean’s critical role in regulating climate, providing oxygen, and supporting billions of people. It is important to note that eating fish is not in line with vegan practices.

SDG 15 involves the protection, restoration, and promotion of the sustainable use of terrestrial ecosystems, the sustainable management of forests, combating desertification, halting and reversing land degradation, and halting biodiversity loss.

Here, we are largely discussing plant-based diets as opposed to vegan diets; the former does not exclude meat or fish, including them in small quantities, with plant-based foods being the primary source of nutrition. The problem with a lot of plant-based diets is that they are in fact UPFs or high in fat, salt, and sugar (HFSS) (a UK government classification); fungi or soy are used to create meat substitutes which can be no healthier than meat itself.

In the context of vegan diet adoption, different factors have been considered, such as taste, religious beliefs, and social and familial environments [154,155,156].

When considering vegan behavior adoption in relation to consumer behavior, we consider different models, such as the stage model of self-regulated behavior change [157], transtheoretical models [158], value attitude behaviors [159], the theory of planned behavior [160], and health belief models [161].

Shah and Thanki Joshi [162] showed that environmental beliefs, health beliefs, and anti-speciesism values exert a positive influence on individuals’ attitudes toward adopting a vegan diet.

**Figure 4:**
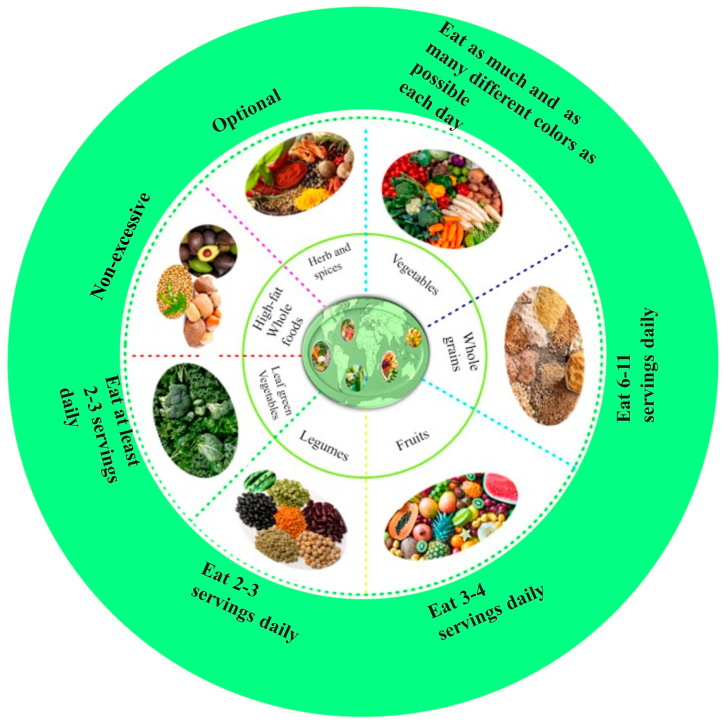
Plant-based food groups and their daily recommended servings.

Moreover, attitude has been reported as a pivotal mediator, connecting social stigma to vegan diet adoption behavior. Figure 4 shows plant-based food groups and their daily recommended servings. The recommended daily servings illustrated in Figure 3 emphasize not only the nutritional adequacy of plant-based diets but also their alignment with broader sustainability and health goals. A balanced inclusion of fruits, leafy greens, legumes, and whole grains ensures essential nutrient intake while reducing environmental impact. The recommendation for daily servings are as follows: fruits—1 serving is 1 piece or ½ cup; leafy green—1 serving is 1 cup raw or ½ cup cooked; legumes—1 serving is ½ cooked legumes or 1 tablespoon seeds; grains—1 serving is ½ cup cooked or 1 slice whole-grain bread [163].

These guidelines therefore serve as a practical framework for individuals, institutions, and policymakers seeking to promote diets that are simultaneously health-promoting, ethically grounded, and ecologically responsible.

## 12. A Paradigm for a Sustainable and Healthy Future in Africa Through the One Health Approach

The One Health framework is designed to create a desired impact in preventing, predicting, detecting, and responding to health threats and enhance the health of human beings, animals, plants, and the environment, as well as concurrently contributing sustainable development. Hence, the One Health joint plan of action (OH-JPA, 2022–2026) developed a guide containing six interdependent action tracks (as shown in Table 3) on how the One Health principles could be applied to strengthen collaboration, communication, capacity building and coordination mutually throughout all sectors and to interface the health concerns for human–animal–plant–environment balance [164]. It is important to note that South Africa is one of the main sources of grain for other countries in the region.

Traore et al. [165] argues that existing health security assessment frameworks show limited scope as well as having limitations in sufficiently considering complex social, political, economic, regulatory, and ecological factors. Therefore, approaches like One Health could be used to develop frameworks that interface among humans, animals, and ecosystems to develop global health security. They concluded that such frameworks should ensure the process of prioritization and building capacity following the core One Health principles. In addition, any added values, trade-offs, and benefits across human, animal, and environmental health systems should be the core interventions and outcomes during assessments. For instance, Zinsstag et al. [166] summarized the advancement and application of One Human–Animal–Environment Health for global health security. They assert that such approaches should be crucial and sustainable to prevent growing risks and hazards, and ensure preparedness, early detection, and investigation. It creates an evidence base to monitor endemic and neglected tropical diseases. Figure 4 depicts the integrated components of the One Health approach. National One Health platforms have been developed as a setup by many sub-Saharan African countries to integrate surveillance and control of zoonotic diseases, food safety and security, antimicrobial resistance, poverty, as well as other health and socioeconomic problems [167]. In Africa, National One Health platforms have gained momentum, providing the capacity to identify prioritization, strengths, weaknesses, opportunities, and gaps during implementation, yet they need improvement [167]. Applying the One Health approach helps to tackle parasitic and vector-borne infections of humans, animals, or both which are of topical relevance to the African continent, ensuring better food security, livelihood, and public health [168]. Zhao et al. [169] conducted a zoonoses assessment of performance in sub-Saharan African countries employing the global One Health index. They reported that this region had high performance for sub-indicators in zoonoses surveillance and response, vector and reservoir interventions, and natural protected areas, indicating that the countries in this region have a specific capability to monitor and prevent or respond to any zoonotic cases.

Today, many challenges such as understaffing, underfunding of institutions, limitations of interdisciplinary cooperation, collaboration, and coordination [170], increasing risks of zoonotic infections [171], raising awareness, commitments, and creating policy influence [172], demanding an integrated set of policy approaches. Farmers’ food safety education, incorporation of regulations, organizations, and multidisciplinary research during the introduction of emerging technologies (genetically modified organisms, nanotechnology, and vertical farming) on food safety [173], etc., have been continued as obstacles to assure the One Health goals in Africa. Table 3 shows summaries of some case studies conducted in the African continent, focusing on the One Health joint plan of action (OH-JPA, 2022–2026) tracks.

The recognition of vegan identity and the principles of social inclusion highlight how individual choices and values intersect with systemic transformations in food systems. At the same time, the One Health approach in Africa illustrates how integrated frameworks can address health, environmental, and food security challenges at regional and global scales. Together, these perspectives demonstrate that sustainable food system transitions require both cultural and behavioral shifts, as well as coordinated institutional and policy responses. The article, therefore, concludes by emphasizing the importance of uniting ethical consumption, technological innovation, governance, and multi-sectoral collaboration to advance resilient, equitable, and sustainable food systems worldwide.

Africa presents a highly heterogeneous landscape with substantial variation across regions in terms of public health capacity, food system infrastructure, and governance mechanisms. Several studies indicate that while East African countries (such as Uganda) have made progress in establishing functional One Health coordination platforms, West and Central Africa continue to face structural barriers, including weak surveillance systems, fragmented veterinary services, and limited laboratory capacity [167,174,175]. Moreover, the burden of zoonotic diseases such as Rift Valley fever, Lassa fever, and trypanosomiasis differs markedly between ecological zones, highlighting the need for region-specific interventions rather than continental generalizations, as reported by Zhao et al. [169]. Trade-offs are also evident: expanding livestock production to address food insecurity can increase greenhouse gas emissions and exacerbate human–wildlife conflict, whereas rapid adoption of advanced biotechnologies may worsen inequality due to limited access among smallholder farmers [167,176]. These disparities underline that One Health implementation in Africa must be tailored, data-driven, and context-specific rather than approached as a uniform continental strategy.

### 12.1. Regional Disparities and Trade-Offs in African Food Systems

African food systems are marked by profound regional disparities stemming from differences in economic prosperity, infrastructure development, health standards, and policy frameworks across countries. These differences not only hinder the transition to sustainable and ethical food systems but also perpetuate inequality and social divisions, particularly when balancing plant-based diets with livestock production under a One Health approach.

Africa’s 54 countries exhibit stark contrasts in gross domestic product (GDP) per capita, agricultural investment, and access to resources, which directly impact food security. For instance, wealthier nations like South Africa (GDP per capita ~USD 6000 in 2025) benefit from advanced irrigation systems and market integration, enabling higher crop yields (e.g., cereals at 4–5 tons/ha), whereas landlocked, low-income countries like Burkina Faso (GDP per capita ~USD 800) face chronic underinvestment, resulting in yields as low as 1 ton/ha [177,178]. These economic disparities translate into uneven standards of living, with urban centers in prosperous regions enjoying better food access via imported goods, while rural populations in poorer countries rely on subsistence farming, which is vulnerable to climate shocks [179,180].

Infrastructure plays a pivotal role in amplifying these gaps. In sub-Saharan Africa (SSA), only about 40% of the population has reliable electricity, and road networks are underdeveloped in many areas, leading to post-harvest losses of up to 30–50% in perishable goods [181]. Countries like Ethiopia and Kenya have invested in transport and ICT infrastructure, improving food distribution and market access, which enhances food security by 0.009–0.02% per infrastructure unit improvement [182,183]. In contrast, nations such as the Democratic Republic of Congo suffer from inadequate water and sanitation systems, exacerbating health issues like malnutrition and stunting, affecting over 40% of children in some regions [182,183,184].

Health standards further underscore these disparities. Prosperous countries with robust policies, such as Rwanda’s universal health coverage, integrate nutrition into public health strategies, reducing undernourishment to below 20%. However, in conflict-affected areas like Somalia or South Sudan, weak health infrastructure leads to higher rates of food-related diseases, with climate change intensifying vulnerabilities through droughts and floods. Policy differences are evident: While the African Union’s Comprehensive Africa Agriculture Development Programme (CAADP) aims for 10% budget allocation to agriculture, only 10 countries meet this target, leaving others with fragmented approaches that fail to address zoonotic risks in livestock systems [185,186].

### 12.2. Trade-Offs in Plant-Based Diets vs. Livestock Production

In the African context, transitioning to plant-based diets offers environmental benefits, such as lower greenhouse gas emissions (plant foods emit <1 kg CO_2_-eq/kg vs. >30 kg for beef), but involves significant trade-offs that intersect with disparities. Livestock, central to livelihoods in pastoral regions (e.g., East Africa), provides high-quality protein and utilizes marginal lands unsuitable for crops, supporting biodiversity and economic resilience. However, intensive livestock farming contributes to deforestation and emissions, creating environmental trade-offs [187,188,189,190].

Nutritionally, plant-based shifts could address micronutrient deficiencies if promoted with fortified foods, but in protein-scarce areas, reducing livestock reliance risks exacerbating hidden hunger without alternatives like algae or pea proteins. Economic trade-offs are pronounced: In livestock-dependent economies like Kenya or Zambia, policies favoring plant-based systems could displace smallholders, increasing unemployment and inequality. Ecosystem trade-offs, such as balancing grazing for soil health against crop expansion, further complicate sustainability in diverse biomes [191,192,193,194].

### 12.3. Impacts on Inequality and Social Division

These disparities foster inequality by concentrating benefits in prosperous urban areas, while rural and low-income populations face food affordability challenges—e.g., healthy diets cost 31–72% more in Africa in comparison with global averages [195]. Social divisions arise from uneven access: in urban areas of SSA, food systems favor imported processed foods, widening the gap with rural communities reliant on traditional livestock, leading to migration and conflict over resources. Gender inequalities compound this, as women in agriculture (40–60% of the workforce) often lack land rights under patriarchal policies [186,191,196]

A rights-based approach, integrating One Health, could mitigate these by prioritizing equitable policies, such as subsidies for neglected species (e.g., guinea fowl) in underserved regions. Circular bioeconomy strategies, like waste-to-feed conversion, offer pathways to reduce trade-offs and promote inclusion [186,190].

Future directions should include harmonized regional policies under the African Continental Free Trade Area to minimize disparities and trade-offs, ensuring ethical food systems benefit all [180,197,198].

**Table 3:** Six interdependent action tracks by the One Health Joint Plan of Action (OH-JPA, 2022–2026) [164] and some related case studies in Africa.

OH-JPA (2022–2026) Action Track		Name of the African Country	Main Focus(es)	Research Outcome(s)	Reference
1	Improving the One Health approach to build stronger health systems	Uganda	One Health approach to health security	Investing in the funding gaps reinforces Uganda’s health security	[175]
		Ethiopia	Implementation of the OH approach	Understaffing, underfunding of institutions, limitation of interdisciplinary cooperation, collaboration, and coordination among animal and human health practitioners are obstacles	[170]
2	Minimizing the widespread threats posed by new and recurring animal-borne diseases	All Africa	Urbanization, armed conflict, and deforestation	Increased risks of zoonotic infections on the environment, animal health, and human health	[171]
			Limitations in the detection of new infectious disease outbreaks in the community, in rapid pathogen identification, and in proactive surveillance systems	Main gaps in public health readiness, detection, and response systems The main paradigm shift is required to develop an effective infrastructure and common frameworks	[199]
3	Manage and eradicate endemic zoonotic, neglected tropical, and vector-borne diseases	African Union member states	Controlling the continental strategy for zoonotic disease	Raising awareness, commitments, and creating policy influence	[172]
			Sustainable and effective strategies for post-elimination control of neglected tropical diseases	Post-elimination control of NTDs remained as challenging	[174]
		Sub-Saharan Africa	Urbanization, fast population growth, increased demand for animal food, and natural habitats invasions	Infectious disease outbreaks are caused by zoonotic pathogens	[176]
			Interventions against neglected tropical diseases	Insecticide resistance, multiplicity of vector species, changes in vector behavior, and cost	[200]
			Vector-borne helminthiases: onchocerciasis, lymphatic filariasis, loiasis, and mansonellosis	Onchocerciasis and lymphatic filariasis have established global elimination programs Loiasis and Mansonellosis have largely been neglected and do not have large-scale control programs	[201]
		Zambia	Zoonotic transmission of vector-borne pathogens in humans	The occurrence of many vector-borne zoonotic pathogens circulating in vectors and animals	[202]
4	Enhancing food safety risk analysis, management, and communication	Sub-Saharan Africa	Food fraud threatens food safety and security	High production costs, weak regulatory systems, cultural practices, and technological limitations	[203]
			Waste management practices by street vendors and factors influencing their mismanagement	A lack of recognition and comprehensive laws and regulations to monitor waste management	[204]
			Effects of emerging technologies (genetically modified organisms, nanotechnology, and vertical farming) on food safety	Requirements of an integrated set of policy approaches, farmers’ food safety education, incorporate regulations, organizations, and multidisciplinary research	[173]
			Meat value chains and prevalent risks	Limitations in policy actors and the incorporation of a participatory approach in the street-vending sector	[205]
		Nigeria and Ghana	Harnessing food safety	Weak enforcement of food safety laws is contributing to complications in the food production chain	[206]
5	Combating the quiet crisis of antimicrobial resistance	Developing countries in Africa	Fundamental conflicts that complicate efforts to control the proliferation of antimicrobial resistance	Antimicrobial resistance policies and actions require balancing the interests of all relevant stakeholders, considering the interests and wellbeing of future generations	[207]
		Sub-Saharan Africa	Antimicrobial resistance’s causes and challenges in implementing prevention measures	Weak antimicrobial resistance surveillance and absence of collaboration, irrational use of antibiotics, poor medicine regulatory systems, lack of infrastructural and institutional capacities, deficiency of human resources, and inefficient infection prevention and control (IPC) practices	[208]
6	Ensuring One Health and environmental integration	Sub-Saharan Africa	Integrated One Health on preventing and managing zoonotic and environmental health threats	Integrating One Health in national agendas and a unified continental framework is required	[209]
			Push-pull technology to sustain vegetable production and maintain soil health and fertility, human and animal nutrition, and food safety	The cropping system could contribute to eradicating zoonotic diseases by incorporating companion plants that fend off disease vectors	[210]
		Nigeria	One Health framework to mitigate cholera outbreaks through integration of human, animal, and environmental health	The One Health framework enables understanding of cholera dynamics and promotes sustainable solutions to deter future outbreaks	[211]

**Figure 5:**
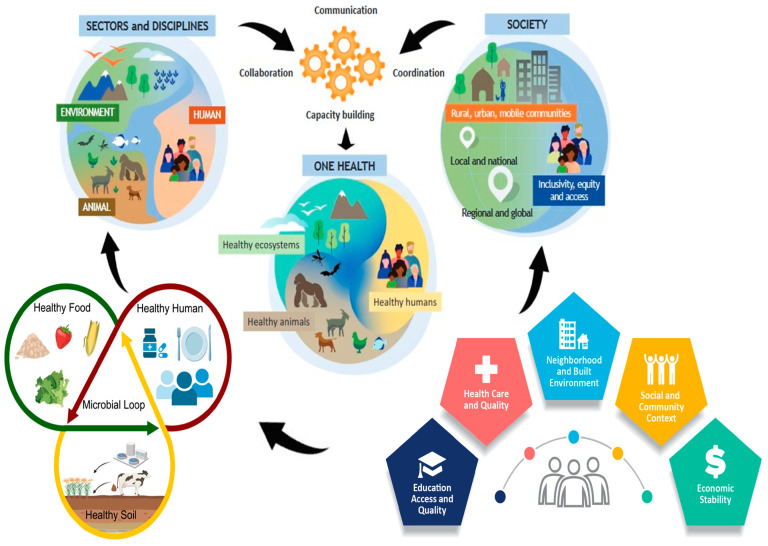
Integrated One Health approach [32,212].

A one Health approach is described through Figure 5 below.

## 13. Conclusions and Future Directions

Sharing nutrition- and environment-related knowledge is increasingly regarded as a strategic component in promoting ethical and sustainable food systems. Rather than being a solely educational activity, it contributes to the transformation of food culture, policy frameworks, and consumer behavior. Although progress has been made, particularly in the integration of environmental goals into dietary guidelines, greater cooperation between academic institutions, governmental bodies, media channels, and civil society remains essential for further advancement. These collaborations are necessary to ensure that science-based messages reach the public in a reliable and comprehensible manner.

In addition, transparency across all stages of food production and distribution should be improved. The implementation of mandatory environmental labeling and disclosure of sourcing practices by companies can support more informed and responsible consumer choices. This can contribute to both individual awareness and market-level change. Moreover, promoting critical engagement with food systems, through food literacy programs and public dialogue, can enable consumers to better understand the broader implications of their dietary decisions. Such awareness may lead to more deliberate choices, reductions in environmental impact, and improvements in long-term public health outcomes.

With the growing availability of digital tools and open-access data, the potential to influence food systems at scale is increasing. The intersection of education, transparent policy, and citizen participation can be viewed as a foundation for achieving food systems that are not only sustainable but also socially just and resilient.

Overall, the evidence indicates that the transformation of food systems cannot be achieved through fragmented or isolated measures. An integrated approach is required, one that brings together ethical consumption, technological innovation, scientific communication, and multi-sectoral governance. Situating sustainability and ethics at the center of food production, policymaking, and everyday practice will enable the development of systems that are resilient and nutritionally adequate, while also equitable and just, ensuring that future food pathways serve both humanity and the planet. Beyond individual components, the article suggests viewing the food system as an interconnected ecosystem in which health, environment, culture, and economics are mutually reinforcing, and underlining that meaningful change depends on systemic integration rather than incremental adjustments.

Plant-based diets is a solution but one should bear in mind that meat proteins also play a key role in human diet covering all essential amino acids in adequate amounts, being significant for growth, preservation of muscles, immune function, and metabolic health. Livestock from a food security perspective transform human-indigestible feedstocks, for instance crop residues, low-quality forages, surplus crops, and organic waste, into high-quality protein, thus improving resource efficiency and decreasing food loss.

Finally, meat also provides higher quality protein than plants (1.4:1) and creates 1 high-quality unit of protein per 1.4 units of plant protein they consume. A topic collection in *Animals* (MDPI) on the “Use of Agricultural Byproducts in Animal Feeding” has also been reported by Caroprese [213].

## Figures and Tables

**Table 1:** Overview of knowledge dissemination channels in nutrition and sustainability.

Method	Key Strengths	Target Audience	Example Tools/Initiatives
Education Systems	Builds lifelong habits and awareness	Students, educators	School gardens, integrated sustainability curricula
Media and Digital Platforms	Fast, broad reach, and cultural influence	General public, youth	Social media campaigns, documentaries
Public Policy and Labeling	Structural change informs purchasing	Consumers, institutions	Eco-labels, national dietary guidelines
Scientific Engagement	Credibility, evidence-based advocacy	Researchers, policymakers	LCA models, citizen science, public lectures

**Table 2:** Processing technologies, commercialization status, and consumption trends for established and emerging plant-based protein sources.

Plant Source	Main Processing Methods	Functional Applications	Commercialization Level	Consumer Acceptance/Trends	Reference
Soy	Soaking, milling, extrusion, fermentation	Meat/dairy analogs, protein isolate	Widely commercialized	High in Asia and Western countries	[137]
Pea	Protein extraction, emulsification, film formation	Protein drinks, meat analogs, emulsifiers	Rapid growth, global brands use it	Growing due to hypoallergenic nature	[138]
Algae (e.g., Spirulina, Chlorella)	Drying, homogenization, protein extraction	Functional foods, supplements, meat analogs	Niche but growing	Moderate; increasing interest as “superfoods”	[139]
Lentils/Legumes	Milling, fermentation, extrusion	Plant protein blends, textured proteins	Well-established	Stable consumption; eco-conscious markets	[140]
Chickpeas	Roasting, fermentation, drying	Hummus, dairy alternatives, baked goods	Expanding	Popular in Middle East and now globally	[141]
Oats	Milling, enzymatic hydrolysis	Oat milk, cereal bars, dairy alternatives	Very high	Very high, especially in oat milk	[142]
Rice protein	Alkaline extraction, filtration	Beverages, protein blends	Mid-stage commercialization	Growing in sports and allergen-free markets	[143]
Hemp	Cold pressing, decortication, protein extraction	Beverages, protein powders, bakery products	Niche, expanding post-legalization	Growing among health-conscious consumers	[144]
Fava Beans	Dehulling, air classification, dry fractionation	Snacks, meat alternatives	Rising	Good acceptance in EU	[145]
Quinoa/Amaranth	Milling, extrusion, puffing	Cereal mixes, meat/dairy alternatives	Early stage	Trendy in functional food segments	[146]
Jackfruit	Minimal	Whole-food meat alternative	Small-scale commercialization	Positive trend in vegan cuisines	[147]
Mushroom/Mycelium	Fermentation, mycelium cultivation	Meat substitutes, umami enhancers	Experimental to emerging	High for health and sustainability appeal	[148]
Duckweed (Lemna)	Wet biomass processing, centrifugation	Protein isolate, smoothies	R&D/Start-up stage	Low; education needed	[149]

## Data Availability

No new data were created or analyzed in this study. Data sharing is not applicable to this article.
